# Marine-Derived Natural Products Against Flaviviruses: Mechanisms, Evidence, and Future Directions

**DOI:** 10.3390/md24080291

**Published:** 2026-08-21

**Authors:** Hyeon Seung Park, Min Seo Heo, Hyuk Nam Kwon, Yo Han Jang, Munhyung Bae, Yun Kwon

**Affiliations:** 1 BK21 FOUR Community-Based Intelligence Novel Drug Discovery Education Unit, College of Pharmacy and Research Institute of Pharmaceutical Sciences, Kyungpook National University, Daegu 41566, Republic of Korea; ss522s@knu.ac.kr (H.S.P.); iulike103@knu.ac.kr (M.S.H.); 2 Department of Biological Sciences, University of Ulsan, Ulsan 44610, Republic of Korea; hnkwon@ulsan.ac.kr; 3 Basic-Clinic Convergence Research Institute, University of Ulsan, Ulsan 44610, Republic of Korea; 4 Vaccine Biotechnology Major, Gyeongkuk National University, Andong 36729, Republic of Korea; yhjh0323@gknu.ac.kr; 5 College of Pharmacy, Gachon University, Incheon 21936, Republic of Korea

**Keywords:** marine-derived natural products, flaviviruses, dengue virus, Zika virus, Japanese encephalitis virus, antiviral agents, stage-of-action evidence, RNA-dependent RNA polymerase

## Abstract

Although flaviviruses, including DENV, ZIKV and JEV, remain important causes of febrile, congenital, and neurological diseases, treatment options remain largely supportive, with limited availability of virus-specific antiviral therapies. Marine organisms and marine-derived microorganisms produce chemically distinct antiviral materials, including sulfated polysaccharides, terpenoids, alkaloids, peptides, cyclodepsipeptides, and polyketides. However, their activities range from preliminary extract-level inhibition to direct biochemical target validation, making mechanistic comparison difficult. This review critically evaluates marine-derived anti-flaviviral agents using two complementary dimensions, the infection stage implicated by experimental assays and the strength of evidence supporting that assignment. DENV evidence is dominated by sulfated algal macromolecules that interfere with adsorption or internalization, whereas ZIKV studies encompass lipophilic algal metabolites, fungal alkaloids, cyclodepsipeptides, and a few target-oriented candidates. Across the field, most reports remain stage-associated rather than target-validated. Cross-study potency comparisons are constrained by differences in virus strains, cell models, assay formats, and treatment schedules. JEV-specific evidence is particularly sparse. Based on the DENV and ZIKV evidence map, we propose concise priorities for JEV-oriented discovery: early compound-level dereplication, parallel cytotoxicity testing, orthogonal confirmation of productive infection, stage-resolved assays, and biochemical or genetic validation of conserved flaviviral targets. This evidence-based framework can help distinguish promising chemical candidate scaffolds from preliminary antiviral signals and guide mechanism-informed development of marine-derived natural products against flaviviruses.

## 1. Introduction

Flaviviruses are enveloped, positive-sense single-stranded RNA viruses that include dengue virus (DENV), Zika virus (ZIKV), Japanese encephalitis virus (JEV), West Nile virus, yellow fever virus, and tick-borne encephalitis virus [1,2,3]. Together, they cause a broad spectrum of substantial febrile, hemorrhagic, congenital, and neurological diseases.

Among flaviviruses, DENV, ZIKV, and JEV remain particularly relevant to public health because they are associated with large regional outbreaks, neurological complications, congenital disease, or severe encephalitis. However, virus-specific therapeutic options remain limited [4,5,6,7]. Although vaccines have been developed for some flaviviral diseases, the clinical management of established DENV, ZIKV, and JEV infections largely relies on supportive care, emphasizing the need for novel antiviral candidates with distinct mechanisms of action [2,6].

Natural products have historically provided structurally diverse bioactive molecules. They remain an important source of chemical matter for drug discovery, especially when conventional synthetic libraries fail to capture complex three-dimensional scaffolds [8,9,10,11,12].

Marine organisms are particularly relevant in this context because they produce chemically distinct metabolites shaped by unique ecological pressures, including halogenated compounds, sulfated polysaccharides, terpenoids, alkaloids, peptides, and polyketides [13,14,15,16,17,18,19]. In antiviral research, marine-derived natural products have been studied against various viruses. Their activities often involve interference with viral attachment, viral entry, viral replication, or virus–host interactions [14,15].

This broader antiviral potential also extends to clinically relevant DNA viruses, particularly herpesviruses such as herpes simplex virus (HSV) [14,20], as well as hepatitis B virus (HBV) [21]. Such activity may partly reflect the distinctive physicochemical and structural properties of marine metabolites. Sulfated polysaccharides from marine algae are highly anionic, multivalent macromolecules that can interact with viral envelope glycoproteins and interfere with their binding to cell-surface glycosaminoglycans, thereby inhibiting viral attachment and entry [20,22]. Marine-derived small molecules can also exert post-entry antiviral effects; for example, the gorgonian-derived diterpenoid junceellolide B was reported to suppress HBV RNA transcription and viral replication [21].

However, for flaviviruses, the available evidence remains uneven. DENV and ZIKV account for most reports of marine-derived extracts, fractions, and isolated compounds, whereas JEV-oriented marine-derived natural product discovery remains comparatively underexplored [6,23]. This review primarily focuses on the mosquito-borne flaviviruses DENV, ZIKV, and JEV. Although marine-derived materials with activity against tick-borne encephalitis virus (TBEV) have also been reported [24,25], TBEV is not discussed in detail because it is a tick-borne flavivirus [26] and therefore falls outside the primary scope of this review.

**Figure 1:**
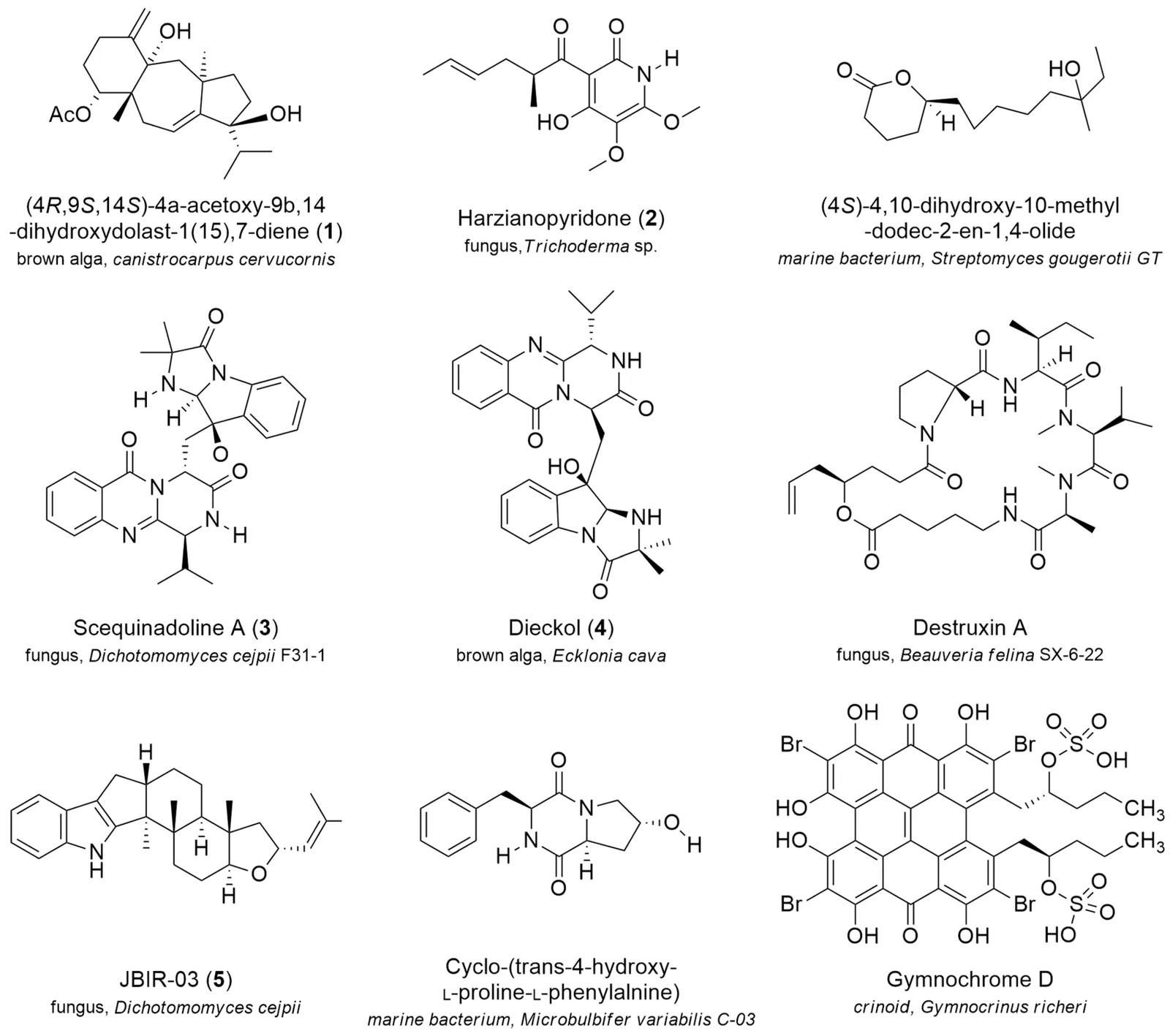
Representative marine-derived anti-flaviviral compounds discussed in this review.

An early study revealed that marine-derived compounds, such as gymnochrome D and isogymnochrome D from the crinoid *Gymnocrinus richeri*, could inhibit DENV-induced cytopathic effects, providing one of the first indications that marine-derived metabolites may serve as anti-DENV candidate scaffolds [27]. Subsequent research further revealed that marine-derived algal sulfated polysaccharides, including galactans and fucoidans, could inhibit DENV infection in a serotype- and host-cell-dependent manner [28,29]. More recently, anti-ZIKV activity has been detected in marine-derived scaffolds such as the diterpene (4*R*,9*S*,14*S*)-4α-acetoxy-9β,14α-dihydroxydolast-1(15),7-diene (**1**) from the brown alga *Canistrocarpus cervicornis*, marine-derived fungal indole alkaloids, marine-derived fungal cyclohexadepsipeptides, and the RdRp-associated marine-related fungal metabolite harzianopyridone (**2**) [30,31,32,33] (Figure 1). Despite these promising examples, many studies remain limited to cell-based antiviral assays, extract or fraction screening, or preliminary time-of-addition experiments. Moreover, direct validation against defined flaviviral targets such as NS2B-NS3 protease, NS3 helicase, or NS5 RNA-dependent RNA polymerase remains limited [6,34]. This limitation is particularly important for studies based on crude extracts or partially purified fractions, as extract-level antiviral activity alone does not identify the active constituent responsible for the observed effect. Chemical complexity and variability in extract composition also make it challenging to standardize activity, compare results across studies, and optimize structure–activity relationships [8]. Therefore, dereplication, isolation, and structural characterization of active constituents are essential for converting marine-derived antiviral hits into reproducible and chemically tractable anti-DENV candidate scaffolds [35,36].

Prior reviews have largely categorized marine-derived antiviral agents according to source organism, chemical class, or target virus. This categorization is descriptively useful but does not clearly distinguish preliminary extract-level inhibition from stage-resolved or target-validated antiviral evidence [23,37,38]. Hence, this review evaluates marine-derived anti-flaviviral agents using two complementary dimensions: (1) the stage of the viral life cycle implicated by the strongest available assay and (2) the strength of evidence supporting that assignment. These dimensions are intentionally separated because a life-cycle stage and a mechanistic certainty level are not equivalent.

## 2. Stage-of-Action Classification Framework

### 2.1. Literature Search and Scope

PubMed, Web of Science, Scopus, and Google Scholar were searched through June 2026 using combinations of the following terms: “marine natural product,” “marine-derived,” “algae,” “marine fungus,” “marine bacterium,” “dengue,” “Zika,” “Japanese encephalitis,” “flavivirus,” and “antiviral.” Primary studies reporting cell-based, biochemical, or in vivo antiviral data were prioritized. Extracts, fractions, and purified compounds were included but explicitly distinguished. Studies exclusively based on molecular docking or molecular dynamics were retained only as hypothesis-generating evidence and were not treated as evidence of experimentally validated antiviral activity.

### 2.2. Dual-Axis Classification, Stage of Action and Evidence Strength

**Figure 2:**
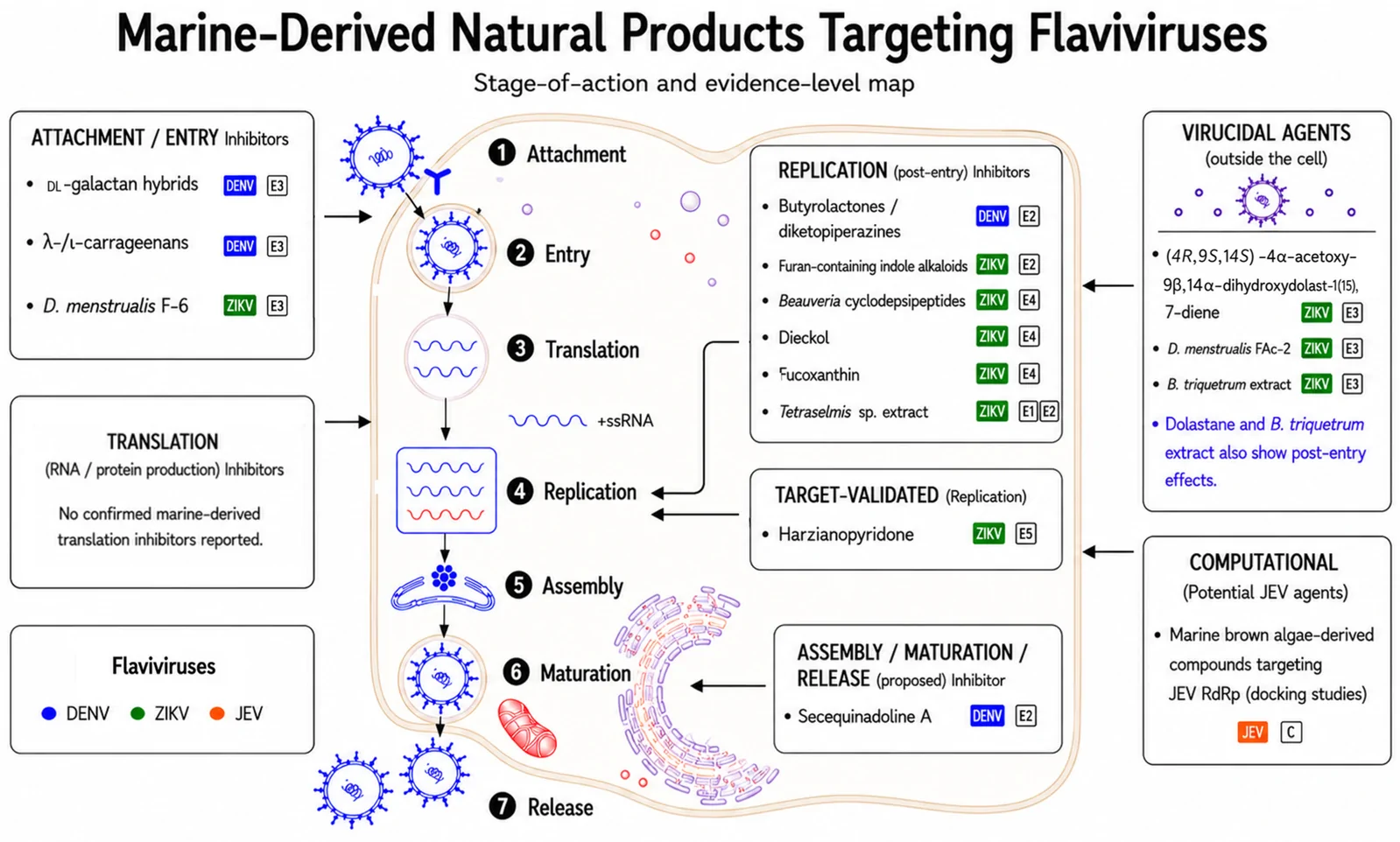
Stage-of-action and evidence-level map of representative marine-derived anti-flaviviral agents across the flavivirus life cycle. Experimentally supported examples are summarized from [20,28,30,31,32,33,44,45,46,47,48,49,50], whereas computationally proposed JEV RdRp candidates are shown separately based on [51].

As shown in Figure 2, the life cycle of flaviviruses comprises attachment, internalization, fusion, uncoating, polyprotein translation and processing, RNA replication, virion assembly, maturation, and release [39,40,41,42]. Structural analysis of the DENV E protein has provided a molecular basis for the low-pH-triggered membrane-fusion step during viral entry [43]. Reported agents were assigned to a specific life-cycle stage only when the strongest available experimental evidence supported the placement. Virucidal activity was treated separately because direct loss of particle infectivity does not necessarily identify a cellular stage of action. When available data could not differentiate between entry, replication, or particle production, the agent was classified as unresolved or broadly suppressing productive infection (Table 1).

Mechanistic certainty was scored independently from stage assignment. Evidence level E1 denotes endpoint activity without compound-level or stage-resolved support. E2 denotes reproducible dose–response activity with cytotoxicity or selectivity data. E3 denotes stage-specific experiments such as adsorption, internalization, virucidal, or time-of-addition assays. E4 denotes orthogonal virological evidence, such as concordant reductions in infectious yield, viral RNA, or viral protein and E5 denotes direct biochemical, biophysical, or genetic target validation. Therefore, a compound can be assigned to a plausible stage but still lack a defined molecular target (Table 2).

### 2.3. Virus-Directed, Potentially Host-Directed, and Mechanistically Unresolved Antiviral Mechanisms

Virus-directed and host-directed antiviral mechanisms represent distinct strategies for suppressing flaviviral infection. Virus-directed agents act directly on viral particles or virus-encoded components, whereas host-directed agents interfere with cellular factors or pathways that are required for efficient viral infection and replication [52]. Because flaviviruses depend extensively on host-cell machinery throughout their life cycle [39,53], host processes such as endoplasmic reticulum (ER)-associated membrane remodeling, lipid metabolism, protein homeostasis, autophagy, and cellular signaling represent potential points of antiviral intervention [39,53,54,55,56].

ER-associated membrane remodeling is particularly important for flaviviral RNA replication. Following infection, flaviviruses reorganize host ER membranes to generate specialized replication organelles that provide a spatially organized environment for viral RNA synthesis. The formation and maintenance of these structures require interactions between viral proteins and host membrane-remodeling and trafficking machinery [39,53]. Lipid metabolism is closely linked to this process because flavivirus infection alters cellular lipid synthesis, transport, and utilization to provide membrane components and metabolic resources required for replication-organelle formation and virion production [40]. Thus, interference with host membrane organization or lipid homeostasis can potentially restrict flaviviral replication without directly targeting a viral protein [39,54].

Protein homeostasis and autophagy constitute additional host processes exploited during flaviviral infection. Viral protein production places substantial demands on cellular protein-folding and degradation systems and can engage ER stress responses, the unfolded protein response, ubiquitin–proteasome pathways, and autophagy. These pathways can influence viral protein production, intracellular membrane dynamics, metabolic homeostasis, and the survival of infected cells. Their pharmacological modulation therefore represents another potential host-directed strategy, although the consequences of autophagy and stress responses can vary depending on the flavivirus, host-cell type, and stage of infection [39,54].

Host cellular signaling and innate immune responses provide additional opportunities for antiviral intervention. Flaviviruses interact with and modulate interferon-associated signaling, inflammatory responses, and other innate immune pathways to establish an intracellular environment permissive for infection [39,55]. Modulating these cellular responses may consequently restrict viral replication by restoring or enhancing host antiviral defenses rather than by directly inhibiting a viral component [55]. This distinction is particularly important when interpreting changes in cytokines or interferon-related markers, because such changes may indicate host-response modulation but do not by themselves establish a specific host molecular target.

Despite the relevance of these host pathways to flaviviral infection, direct validation of specific host targets remains limited among the marine-derived anti-flaviviral agents reviewed here. Therefore, evidence of altered host responses should be interpreted cautiously unless supported by direct genetic, biochemical, or target-engagement studies. The distinction among virus-directed, potentially host-directed, and mechanistically unresolved marine-derived agents is summarized in Table 3.

### 2.4. Limitations of Stage Assignment in Marine-Derived Natural Product Studies

Stage-of-action assignments should be interpreted as evidence-based classifications rather than definitive mechanistic conclusions. Assays such as time-of-addition experiments can identify the stage at which antiviral activity is most apparent, but such assays alone cannot establish a direct molecular target [57]. Likewise, reductions in viral RNA, viral protein expression, or infectious virus production may result from direct inhibition of viral processes, modulation of host-cell pathways, or indirect effects associated with cytotoxicity. Furthermore, comparisons of antiviral potency across studies remain challenging because of differences in virus strains, cell lines, multiplicities of infection, treatment schedules, and assay endpoints [58,59]. Accordingly, the framework proposed here is intended to organize the available evidence according to the strength of mechanistic support rather than to imply equivalent mechanistic certainty across studies.

## 3. Marine-Derived Inhibitors of Viral Attachment, Adsorption, and Entry

### 3.1. Sulfated Galactans and Carrageenans as DENV Entry Inhibitors

Marine-derived natural products that interfere with viral attachment, adsorption, or entry represent an important class of anti-flaviviral agents, because inhibition at these early stages can prevent subsequent viral genome replication and progeny virus production [23,28] (Table 1). Among marine-derived anti-DENV agents, sulfated polysaccharides from marine algae provide some of the strongest and most consistent evidence for inhibition of viral attachment and entry [23,28,29].

Early research on dl-galactan hybrids from the red seaweed *Gymnogongrus torulosus* demonstrated potent inhibition of DENV-2 in Vero cells. These dl-galactan hybrids inhibited DENV-2 plaque formation with IC_50_ values ranging from 0.19 to 1.7 µg/mL. Moreover, they lacked detectable cytotoxicity in stationary and actively dividing cells, and achieved complete inhibitory activity when present during the viral adsorption period [20]. One proposed virus-directed mechanism of sulfated polysaccharides involves electrostatic interactions with positively charged regions of the DENV envelope (E) protein [60,61]. Consistent with this mechanism, DENV has been shown to attach to heparan sulfate-containing proteoglycan on host cells, and soluble heparin or heparan sulfate can inhibit viral infection [61]. Acting as highly sulfated polyanionic mimics of cell-surface glycosaminoglycans, sulfated polysaccharides may therefore competitively interfere with DENV attachment to heparan sulfate-containing host-cell receptors, thereby reducing viral adsorption and subsequent entry [28,60,61,62].

Consistent with this entry-focused mechanism, λ-/ι-carrageenans were found to inhibit DENV-2 and DENV-3 multiplication in Vero and HepG2 cells, with EC_50_ values ranging from 0.14 to 4.1 µg/mL depending on carrageenan type, virus serotype, and host-cell system. In Vero cells, no cytotoxicity was detected at concentrations up to 1000 µg/mL (CC_50_ > 1000 µg/mL), and the reported SI values ranged from 2500 to 6666 for DENV-2 and from 244 to 500 for DENV-3 [28] (Table 3).

That study further indicated that the inhibitory effect involved both viral adsorption and subsequent nucleocapsid internalization and uncoating. However, no inhibition was detected when the entry process was bypassed by DENV-2 RNA transfection [28]. A broader assessment of sulfated polysaccharides from red, brown, and green seaweeds confirmed that DENV-2 was the most susceptible among the four DENV serotypes, with IC_50_ values ranging from 0.12 to 20 µg/mL [63]. In that comparative study, antiviral potency was associated with sulfate content, sulfate position, sugar composition, and molar mass, and the major antiviral effect was again noted during DENV-2 adsorption and internalization [63,64].

Collectively, these findings support an entry-directed mechanism for marine sulfated polysaccharides, with the strongest effects occurring during viral adsorption and internalization [23,28,61,65]. The substantial variation in antiviral potency among these polysaccharides further indicates that activity depends on specific structural features [61,65], which are discussed in the following section.

### 3.2. Structural Determinants of Antiviral Activity in Marine Sulfated Polysaccharides

Although systematic DENV-specific SAR studies remain limited, available analyses of marine sulfated polysaccharides indicate that antiviral potency is governed by multiple interdependent structural features rather than by sulfate content alone [66,67,68]. Sulfate density and sulfation pattern are particularly important because they determine the spatial distribution of negative charges that can mediate electrostatic and multivalent interactions with positively charged regions of viral surface proteins [66,67]. Molecular weight can further modulate antiviral activity by affecting chain length, multivalent binding, solubility, and accessibility to viral targets, although the relationship between molecular weight and potency is not necessarily linear [22,68].

Monosaccharide composition, glycosidic linkage, branching, and overall polysaccharide conformation may additionally influence binding affinity and target accessibility [62,63,64].

Collectively, these observations suggest that the entry-inhibitory activity of marine sulfated polysaccharides reflects the combined effects of charge density, sulfation pattern, molecular size, and three-dimensional chain architecture, providing a structural basis for the future optimization of glycan-based anti-flaviviral agents [22,63,68].

### 3.3. Marine Algal Extracts with Early-Stage Anti-DENV Activity

Marine-derived seaweed extracts have also been screened using cell-based assays against all four DENV serotypes. Koishi et al. established an in situ ELISA assay in DENV-infected Huh7.5 cells with an average signal-to-background ratio of 7.2 and a Z-factor of 0.62. They screened 15 marine-derived seaweed extracts at their maximum non-toxic concentrations [69]. Eight of these seaweed extracts reduced infection caused by at least one DENV serotype. Four extracts, derived from *C. cervicornis*, *Padina gymnospora*, *Palisada perforata*, and *Caulerpa racemosa*, were selected for further evaluation. Time-of-addition experiments suggested that these active extracts act at an early stage of DENV infection, such as viral binding or internalization, rather than at later stages of replication [69] (Table 3).

### 3.4. Marine-Derived Inhibitors of ZIKV Adsorption and Entry

In the ZIKV literature, the brown seaweed *Dictyota menstrualis* yielded active fractions with different stage-of-action profiles. The F-6 fraction exhibited a CC_50_ value of 592 µg/mL, an EC_50_ value of 2.80 ± 0.10 µg/mL, and a selectivity index (SI) of approximately 211. Moreover, mechanistic assays suggested that the F-6 fraction inhibited viral adsorption [46] (Table 3).

Because *D. menstrualis* fractions are chemically complex and contain multiple constituents, these findings should be interpreted as stage-of-action evidence rather than as direct validation of a defined molecular target. In particular, inhibition during viral adsorption does not by itself establish direct interaction with a specific ZIKV envelope protein or host-cell attachment factor. Nevertheless, these results demonstrate that marine algal materials can provide early-stage anti-ZIKV activity and extend the entry-directed antiviral evidence beyond the sulfated polysaccharides more extensively characterized against DENV [46].

### 3.5. Translational Implications of Entry-Directed Marine Antivirals

Collectively, the DENV and ZIKV studies indicate that marine-derived materials can interfere with early stages of flaviviral infection, including viral attachment, adsorption, and internalization [20,28,46,67]. Among these agents, sulfated galactans, carrageenans, and related polysaccharides provide particularly consistent evidence for an entry-directed mode of action, as their antiviral effects are most pronounced during viral adsorption or early infection [20,28].

However, the physicochemical properties of sulfated polysaccharides may also limit their translational potential. High molecular weight, structural heterogeneity, inter-batch variability, and limited conventional drug-likeness may restrict their development as conventional orally available therapeutics [22]. Accordingly, these macromolecules may be particularly useful as mechanistic probes of flaviviral entry, prophylactic or topical antiviral candidates, or structural templates for the development of simplified polyanionic inhibitors [20,28]. In contrast, chemically complex algal extracts and fractions showing early-stage anti-DENV or anti-ZIKV activity require further dereplication, isolation, structural characterization, and compound-level mechanistic validation [35,36,46].

These findings are also relevant to JEV-oriented discovery. Because marine-derived antiviral evidence against JEV remains limited, attachment, adsorption, and internalization assays provide a practical starting point for determining whether entry-directed mechanisms identified in DENV and ZIKV studies extend to JEV [6,70,71,72] (Table 1).

## 4. Marine-Derived Inhibitors with Virucidal and Early Post-Entry Effects

### 4.1. (4R,9S,14S)-4α-Acetoxy-9β,14α-Dihydroxydolast-1(15),7-Diene and Lipophilic Algal Metabolites

In contrast to the literature on the DENV entry-inhibitors, which is dominated by sulfated polysaccharides, studies on anti-ZIKV marine-derived agents include a broader set of lipophilic extracts, algal fractions, terpenoids, fungal alkaloids, cyclodepsipeptides, and other small-molecule scaffolds [30,31,32,73,74,75]. One of the most extensively characterized marine algal examples is the brown seaweed *C. cervicornis*, whose crude extract and isolated (4*R*,9*S*,14*S*)-4α-acetoxy-9β,14α-dihydroxydolast-1(15),7-diene (**1**) were evaluated for their inhibitory effects on ZIKV replication in Vero cells [30] (Figure 1).

The crude extract of *C. cervicornis* inhibited ZIKV replication with an EC_50_ value of 2.15–3.3 µg/mL, depending on the reported assay summary. It also exhibited low cytotoxicity with a CC_50_ value of 438 µg/mL, corresponding to an SI of approximately 203 [30] (Table 3).

The isolated compound **1** exhibited stronger anti-ZIKV activity than the crude extract, with an EC_50_ value of 0.75–0.95 µM, a CC_50_ value of 935 µM, and an SI of approximately 1246, making it one of the most selective marine-derived ZIKV inhibitors reported so far [30]. In the same study, compound **1** exhibited partial virucidal activity against ZIKV, reducing viral infectivity by approximately 64% at 10 µM. It retained its antiviral activity when added up to 8 h post-infection, suggesting that it acts at both early and post-entry stages of the viral replication cycle [30]. Thus, compound **1** is classified as an E3 virucidal/post-entry candidate rather than as a target-defined inhibitor [30].

### 4.2. ZIKV-Active Algal Fractions and Red Seaweed Extracts with Multi-Stage Activity

The FAc-2 fraction of *D. menstrualis* exhibited an EC_50_ value of 0.81 ± 0.04 µg/mL, a CC_50_ value of 482 µg/mL, and an SI of 595.06. Mechanistic assays suggested that this fraction possessed virucidal potential, whereas the F-6 fraction described above inhibited viral adsorption [46]. These different stage-of-action profiles suggest that chemically distinct constituents within marine-derived algal fractions can interfere with different stages of ZIKV infection.

The red seaweed *Osmundaria obtusiloba* has also been reported to inhibit ZIKV replication, with its ethanolic extract showing a CC_50_ value of 525 µg/mL and an EC_50_ value of 1.82 µg/mL [76]. However, as this evidence is mainly based on extract-level antiviral activity, the active constituents responsible for ZIKV inhibition and the precise stage of action remain unclear. Therefore, this extract should be interpreted as a replication-associated or productive infection-suppressing candidate rather than as a target-defined inhibitor until purified compounds are obtained and additional mechanistic assays are performed [76]. A related red seaweed study assessed the dichloromethane extract of *Bryothamnion triquetrum* and reported potent anti-ZIKV activity in Vero cells, with an EC_50_ value of 1.38 ± 0.28 µg/mL, a CC_50_ value of 400 ± 13.5 µg/mL, and an SI of 289.85 [47]. The *B. triquetrum* extract exhibited only moderate virucidal activity, inhibiting approximately 40% plaque formation at 10 µg/mL. However, time-of-addition assays revealed a reduction of approximately 5 log_10_ PFU/mL when the extract was added at 0–2 h postinfection and at least 2 log_10_ PFU/mL when it was added at 6–12 h postinfection [47]. These results suggest that the *B. triquetrum* extract can inhibit both early and later stages of ZIKV replication rather than acting solely as a direct virucidal agent.

### 4.3. Distinguishing Virucidal Effects from Post-Entry Inhibition

Virucidal activity, entry inhibition, and post-entry inhibition are frequently difficult to distinguish in extract-based studies. A virucidal assay can reveal that a sample directly reduces particle infectivity, whereas a time-of-addition assay can reveal whether activity is limited to the inoculation period or is retained after infection is established [30,47].

In chemically complex marine-derived extracts, apparent multistage activity may involve multiple active constituents rather than a single compound acting at several stages of the viral life cycle. Therefore, active fractions such as the *D. menstrualis* FAc-2 fraction or *B. triquetrum* dichloromethane extract should be considered mechanistically suggestive but not target-defined until purified constituents are identified and orthogonal assays are performed [46,47].

## 5. Marine-Derived Inhibitors Affecting Viral RNA Replication and Protein Production

### 5.1. Marine-Derived Bacterial Butyrolactones and Diketopiperazines Against DENV

Although small molecules from marine microorganisms are less extensively studied than algal polysaccharides, they offer chemically defined scaffolds for lead optimization. Lin et al. isolated butyrolactone- and diketopiperazine-type metabolites from *Streptomyces gougerotii* GT and *Microbulbifer variabilis* C-03 and assessed their efficacy against DENV-2 [48].

The metabolites 4*S*,10-dihydroxy-10-methyl-dodec-2-en-1,4-olide, cyclo-(l-proline-l-leucine), cyclo-(trans-4-hydroxy-l-proline-l-leucine), and cyclo-(trans-4-hydroxy-l-proline-l-phenylalanine) inhibited DENV-2 replication with EC_50_ values of 21.2, 16.5, 12.3, and 11.2 µM, respectively. Among these metabolites, cyclo-(trans-4-hydroxy-l-proline-l-phenylalanine) exhibited the strongest activity, followed by cyclo-(trans-4-hydroxy-l-proline-l-leucine). As compound-specific CC_50_ values, selectivity indices, and direct target data remain limited, these metabolites are classified as E2 replication-associated hits [48].

### 5.2. Marine Fungal Indole Alkaloids Against ZIKV

Marine-derived fungi have produced defined anti-ZIKV small molecules, expanding the chemical space beyond algal extracts and diterpenoids [31,32]. In one study, supplementation of a marine-derived *Fusarium* sp. L1 culture with L-tryptophan induced the production of indole alkaloids, among which fusaindoterpene B, JBIR-03 (**5**), and 1,2-bis(1H-indol-3-yl)ethane-1,2-dione inhibited ZIKV in a standard plaque assay with EC_50_ values of 7.5, 4.2, and 5.0 µM, respectively [31]. These active indole alkaloids did not show significant cytotoxicity against A549 cells under the assay conditions, supporting their potential as marine fungal anti-ZIKV hits. Because the primary readout was plaque reduction and compound-specific CC_50_ and target-level data were limited, these metabolites are classified as E2 cellular antiviral hits, with their precise stage of action remaining unresolved [31].

### 5.3. Cyclodepsipeptides and Suppression of ZIKV RNA/NS5 Protein Production

Another study on marine-derived fungal agents assessed *Beauveria felina* SX-6-22 and identified 30 cyclohexadepsipeptides, including destruxins, isaridins, and isariins. Many of these cyclohexadepsipeptides reduced ZIKV RNA levels and NS5 protein production in ZIKV-infected A549 cells [32]. These results can be discussed at the interface between RNA replication and viral protein production: the reduction in viral RNA supports a replication-associated effect, whereas reduced NS5 protein levels indicate the suppression of viral protein accumulation but do not distinguish whether the compound directly acts on viral RNA synthesis, protein translation, protein stability, or an upstream host-cell process [32,37].

This finding is particularly relevant because it connects anti-ZIKV activity with nonribosomal peptide biosynthesis, providing a biosynthetic entry point for future engineering of cyclodepsipeptide analogs. All cyclohexadepsipeptides evaluated for cytotoxicity showed CC_50_ values > 100 µM in uninfected A549 cells, whereas the active congeners significantly reduced ZIKV RNA levels and NS5 protein production at 10 µM [32]. However, compound-specific EC_50_ values were not reported; therefore, SI values could not be determined for the individual active congeners [32] (Table 3).

### 5.4. Scequinadoline A and Possible Suppression of Productive DENV Infection

Scequinadoline A (**3**), an alkaloid isolated from the marine fungus *Dichotomomyces cejpii* F31-1, was found to exhibit anti-DENV activity in cell-based assays [44]. In contrast to sulfated polysaccharides, which are mainly considered attachment- or entry-associated inhibitors, compound **3** is proposed as a post-entry or productive infection-suppressing candidate because the available evidence is not centered on viral adsorption or internalization [44]. However, existing data do not clearly distinguish whether the observed reduction in DENV activity is attributed to the inhibition of viral RNA synthesis, suppression of viral protein production, impairment of virion assembly or maturation, reduced release of infectious particles, or indirect host-cell effects. Therefore, compound **3** should be conservatively classified as a post-entry or productive infection-suppressing agent with an unresolved stage of action, rather than as a target-validated inhibitor [44]. This example illustrates a recurring limitation in the literature on marine-derived anti-flaviviral agents: cellular antiviral activity can identify promising chemical scaffolds; however, additional stage-resolved virological assays and biochemical validation are warranted before a precise stage of action or molecular target can be assigned [37,44].

### 5.5. Phlorotannins, Chlorophyll-Related Extracts, and Replication-Associated Readouts

An 80% ethanol extract of *Ecklonia cava* and its major phlorotannin dieckol (**4**) were reported to reduce ZIKV titers and ZIKV mRNA levels in Vero E6 cells. They also suppressed the expression of inflammatory markers such as TNF-α and IFIT in the infected cells [49] (Figure 1).

As these readouts include both viral markers and host inflammatory responses, further experiments are warranted to determine whether the observed antiviral effect reflects direct inhibition of a viral target or an indirect host-mediated effect.

Therefore, these findings should be conservatively described as the suppression of ZIKV replication-associated readouts rather than as direct viral target inhibition [37,49]. Fucoxanthin, an edible carotenoid purified from the brown alga *Sargassum siliquastrum*, also reduced infectious ZIKV particle production and downregulated NS1 mRNA expression in Vero E6 cells. Docking-based analyses further suggested interactions with the ZIKV envelope protein, NS3, and RdRp. Nevertheless, direct biochemical target validation remains necessary [45]. As *E. cava* is an edible brown alga abundant in Korea and Japan, this finding may be useful not only as an anti-ZIKV example but also as a regional marine-derived natural product case that strengthens the relevance of East Asian marine-derived algal resources [49].

Marine microalgae have also emerged as a source of potential anti-ZIKV agents. In particular, chlorophyll extracts from *Tetraselmis* sp. were reported to suppress ZIKV infection, highlighting photosynthetic marine microorganisms as another underexplored source of antiviral metabolites [50]. These examples should be interpreted with caution. Unless viral RNA replication is directly separated from viral entry and release, these findings are best described as the suppression of viral RNA, viral titer, viral protein, or productive infection rather than definitive inhibition of replication.

## 6. Experimentally Supported and Computationally Proposed Targets

### 6.1. Harzianopyridone, an Experimentally Supported ZIKV RdRp-Associated Scaffold

Beyond extracts and classical natural product fractions, marine-derived inhibitors with direct target validation are particularly valuable because flaviviral genome replication depends on viral NS5 RNA-dependent RNA polymerase, an essential enzyme responsible for the synthesis of viral RNA after entry and polyprotein processing [2,56]. Flaviviral polymerases represent attractive antiviral targets because they are directly required for genome replication and can provide a clearer mechanistic endpoint than broad cell-based inhibition alone [7,56]. Harzianopyridone (**2**) has recently been reported as a marine-derived natural product that exhibits direct anti-ZIKV activity by targeting RNA-dependent RNA polymerase [33]. Compound **2** was found to inhibit ZIKV replication with EC_50_ values ranging from 0.46 to 2.63 µM across multiple cellular models. Moreover, it exhibited no obvious cytotoxicity, with CC_50_ values exceeding 45 µM. The combination of low-micromolar antiviral activity and low apparent cytotoxicity is important for lead evaluation because it suggests that the observed inhibition is less likely to result from nonspecific loss of host-cell viability and provides a basis for estimating selectivity relative to other marine-derived anti-ZIKV candidates [33]. Unlike prior marine-derived ZIKV studies that were mainly based on cellular inhibition or time-of-addition experiments [30,31,32,47], compound **2** was reported to interact with ZIKV RNA-dependent RNA polymerase and suppress polymerase activity [33]. This provides stronger mechanistic support than plaque or RNA reduction alone [33]. Nevertheless, the exact strength of the target claim should be interpreted based on the biochemical assay design, binding method, and availability of resistance or mutational evidence [33,37]. This distinction is crucial because most marine-derived flaviviral inhibitors have been mainly characterized by cell-based assays rather than direct biochemical target validation [23,37,38,77].

### 6.2. Computationally Prioritized Marine-Derived Compounds Targeting JEV RdRp

Although studies on JEV-oriented marine-derived natural products remain scarce, compounds derived from marine brown algae have been computationally studied as putative binders of JEV RNA-dependent RNA polymerase [51]. These candidates should be described as computationally prioritized scaffolds rather than JEV inhibitors until biochemical enzyme assays and cell-based antiviral assays are performed. Although docking and molecular dynamics can support hypothesis generation and scaffold prioritization, they do not demonstrate target engagement or antiviral potency, selectivity, or activity in infected cells. Therefore, computational-only evidence is ranked below experimentally supported cellular and biochemical evidence in this review.

### 6.3. Criteria for Direct Target Validation

**Figure 3:**
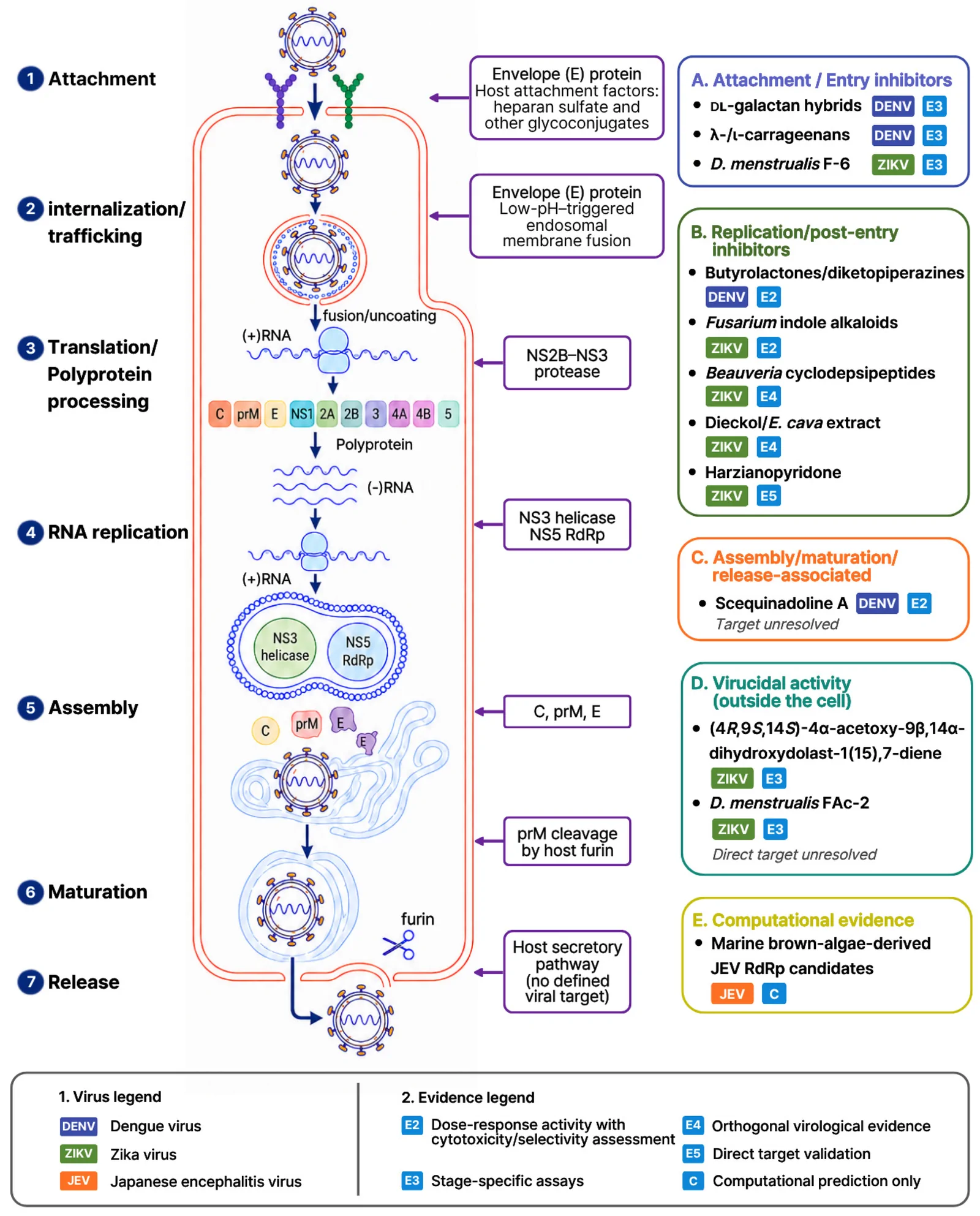
Target-oriented summary of representative marine-derived anti-flaviviral agents, their associated viral proteins, implicated life-cycle stages, and evidence levels.

A major distinction between activity-guided and mechanism-guided discovery is whether an antiviral hit can be linked to a defined viral or host target. In the literature on marine-derived anti-flaviviral agents, such evidence remains rare. Compound **2**, which targets ZIKV RdRp, represents one of the best recent examples. This rarity is likely because many marine-derived studies are conducted with complex extracts or fractions, limited quantities of purified metabolites, and cell-based screening systems, while direct target validation requires additional purified compounds, recombinant viral enzymes, binding assays, or genetic tools that are not always available during early natural product discovery [33,37]. Most other studies rely on cell-based endpoints, such as CPE inhibition, plaque reduction, viral yield reduction, viral RNA reduction, time-of-addition assays, adsorption assays, or virucidal assays. Although these assays are useful for identifying active extracts and compounds, they do not necessarily distinguish between direct antiviral activity, cytostatic effects, host-directed mechanisms, inhibition of viral entry, suppression of replication, or impairment of virion assembly and release. Therefore, the next step after initial activity screening should involve stepwise validation using purified compounds, dose–response confirmation, cytotoxicity and selectivity assessment, time-resolved viral RNA or protein measurements, and target-focused assays (such as enzyme inhibition assays, binding analysis, or resistance/mutational studies) [30,44,47,67]. For target-level validation, the most relevant viral proteins include NS2B-NS3 protease, NS3 helicase, and NS5 RNA-dependent RNA polymerase. All these proteins are conserved enzymatic components of the flaviviral replication machinery. NS2B-NS3 protease cleaves the viral polyprotein to produce functional viral proteins, NS3 helicase unwinds viral RNA structures during replication, and NS5 RNA-dependent RNA polymerase synthesizes new viral RNA genomes [78,79,80,81]. This target-oriented emphasis is also consistent with broader antiviral discovery against flaviviruses, with entry, fusion, polyprotein processing, helicase activity, and NS5-mediated RNA synthesis being repeatedly highlighted as intervention points across DENV, ZIKV, JEV, and related flaviviruses. A recent DENV drug-development study targeting the NS3–NS4B interface further illustrates how molecular target identification, resistance analysis, and preclinical in vivo validation can strengthen mechanism-based antiviral development [82]. This strategy has subsequently advanced to a phase 2a randomized controlled human infection study of mosnodenvir, providing clinical proof of concept that NS3–NS4B inhibition can reduce DENV-3 infection when used prophylactically [83]. Following target validation, promising candidates should be advanced through translational steps, including confirmation in relevant cell models, selectivity and cytotoxicity assessments, target engagement or resistance studies, pharmacokinetic and toxicity profiling, and lead optimization, in order to improve potency, stability, and developability [7,37]. An overview of representative marine-derived anti-flaviviral agents, their implicated life-cycle stages, associated viral proteins, and corresponding evidence levels is provided in Figure 3.

## 7. Priorities for JEV-Oriented Marine Natural Product Discovery

### 7.1. Evidence Gap and Transferable Hypotheses

JEV remains markedly underrepresented relative to DENV and ZIKV in the literature on marine-derived natural products. This limited evidence includes sulfated polysaccharides and computationally proposed polymerase-binding compounds. Among the experimentally evaluated marine-derived materials, carrageenan inhibited JEV infection with an EC_50_ of 15 µg/mL and a CC_50_ > 200 µg/mL in WRL68 cells, corresponding to an SI > 13.3 based on the reported values. Stage-resolved assays further suggested that this activity was primarily associated with inhibition of viral attachment and cellular entry [72]. Sulfated polysaccharide extracts from the green alga *Ulva lactuca* also showed concentration-dependent anti-JEV activity and interference with viral adsorption, although a fitted EC_50_ value was not reported [71] (Table 4). However, few purified molecules are supported by integrated dose–response, selectivity, stage-of-action, and target-level evidence [51,70,71,72]. This gap should be treated as an opportunity for hypothesis testing and not as evidence that DENV- or ZIKV-active compounds will necessarily inhibit JEV [6,84,85,86]. Nevertheless, chemically defined small molecules with activity across multiple flaviviruses provide precedent for testing shared antiviral scaffolds against JEV [87].

DENV studies justify evaluating sulfated polysaccharides in JEV attachment and internalization assays. This rationale is further supported by genetic evidence implicating heparan sulfate proteoglycan metabolism and sulfation in JEV entry [88]. Translation across viruses should be experimentally evaluated because of differences in entry factors, replication kinetics, cell tropism, and assay conditions [20,28,30,31,32,48].

### 7.2. Assay Sequence and JEV-Specific Considerations

Primary screening should pair infectious JEV inhibition with parallel host-cell viability assessment, followed by dose–response determination of EC_50_, CC_50_, and SI. Hits should then be confirmed using at least one orthogonal virological readout, such as plaque- or focus-forming assays, infectious virus yield, RT-qPCR, or viral protein detection. Stage-resolved experiments should be selected according to the chemical class: attachment and internalization assays for sulfated macromolecules, virucidal and time-of-addition assays for lipophilic fractions, and replicon or subgenomic RNA assays for post-entry small molecules [6,28,89]. JEV-oriented validation should preferentially incorporate neurorelevant models where feasible because JEV infects neural progenitor cells, human microglial cells, and developing cortical organoids. Moreover, antiviral responses may substantially differ from those observed in conventional epithelial cell lines [90,91,92]. These neurorelevant models are biologically important because JEV infection can reduce neural progenitor cell proliferation and viability, while developing cortical organoids exhibit maturation-dependent differences in viral susceptibility and antiviral immunity [90,91]. JEV-oriented validation should also incorporate blood–brain barrier (BBB) and multicellular neurovascular models. In a human brain microvascular endothelial cell–astrocyte coculture model, JEV infection increased barrier permeability and induced a strong pro-inflammatory endothelial response [93]. Primary human brain microvascular endothelial cells were also shown to support JEV entry through a Src–ezrin–caveolin-1-dependent endocytic pathway [94]. More recent studies demonstrated that JEV-associated inflammatory signaling increased BBB permeability through hypermethylation of the *Afdn* promoter and downregulation of AFDN, whereas JEV-associated human microglia induced endothelial cell death in a receptor-independent coculture system [95,96].

More recently, spatial transcriptomic analysis further highlighted vasculature-centered cellular interactions associated with JEV progression, reinforcing the relevance of the neurovascular microenvironment in JEV pathogenesis [97].

Collectively, these findings support the inclusion of BBB endothelial cells, astrocytes, and microglia alongside neuronal and organoid models when prioritizing JEV-active compounds. For candidates intended to treat established encephalitic infection, antiviral potency in conventional cell culture alone is unlikely to predict therapeutic utility. Effective JEV therapeutics must achieve sufficient exposure within the central nervous system (CNS), which requires consideration of blood–brain barrier penetration, CNS pharmacokinetics, brain exposure and retention, and the relationship between plasma and brain concentrations [98,99]. These requirements may be particularly challenging for high-molecular-weight or highly polar marine-derived compounds with limited tissue penetration. In addition, treatment of neuroinvasive disease differs from treatment of peripheral viral infection because substantial neuronal injury and neuroinflammation may already be present by the time encephalitic symptoms become clinically apparent. Therefore, JEV-oriented candidates should ultimately be evaluated not only for antiviral potency and systemic safety but also for CNS exposure, neurotoxicity, and efficacy in neurorelevant infection models that better reproduce the cellular and barrier environments encountered during encephalitic disease [98,99].

### 7.3. Compound Identification and Target Confirmation

Dereplication, feature-based molecular networking, and comparison with community-curated spectral libraries can help link antiviral activity to metabolite families, identify known compounds, and prioritize chemically distinct features before extensive isolation [100,101,102,103].

Purified compounds that retain activity in replication-focused assays should be evaluated against conserved JEV enzymes, including NS2B-NS3 protease, NS3 helicase, and NS5 RNA-dependent RNA polymerase. A robust target claim should ideally combine cellular antiviral activity with direct biochemical inhibition or biophysical binding and, where feasible, structural analysis, resistance selection, or mutational evidence [56,104,105]. The workflow summarized in Table 5 is intended as a decision-making sequence rather than a requirement that every preliminary extract be subjected to all assays.

The values in Table 4 are intended for contextual orientation only. They should not be interpreted as a rank-order potency comparison because the virus strain or serotype, cell model, multiplicity of infection, treatment schedule, endpoint, and reporting unit differ across studies. Macromolecular extracts or polysaccharides are also not directly comparable with purified small molecules. Considering these constraints, selected marine-derived candidates show sufficient potency or selectivity to warrant mechanism-guided follow-up. However, their therapeutic potential cannot be inferred without standardized assays, in vivo efficacy studies, pharmacokinetic assessments, toxicity assessments, and formulation data [7,37].

## 8. Translational Challenges and Emerging Technologies in Marine-Derived Antiviral Discovery

### 8.1. Translational and Clinical Development Challenges

Although many marine-derived compounds exhibit promising in vitro biological activity, their translation into clinically useful therapeutics remains challenging. Marine-derived compounds may face pharmacokinetic limitations related to absorption, distribution, metabolism, and elimination, and formulation strategies may therefore be required to improve their bioavailability and systemic exposure [106,107]. Li et al. specifically showed that the marine sulfated polysaccharide propylene glycol alginate sodium sulfate was poorly absorbed after oral administration, whereas an enteric nanoparticle formulation significantly improved its relative bioavailability.

For chemically defined small molecules, subsequent development requires characterization and, where necessary, optimization of pharmacokinetic properties, metabolic stability, and systemic safety. In addition, reliable supply can represent a major challenge for scarce marine natural products. For example, the limited availability of bryostatin 1 had constrained research and clinical development until a scalable synthetic route capable of supplying clinically relevant quantities was developed [108].

Structural and compositional heterogeneity, batch-to-batch variability, formulation, and manufacturing consistency are additional concerns when complex natural extracts or mixtures are advanced toward clinical development. Regulatory considerations for complex natural-product mixtures likewise emphasize adequate characterization, manufacturing consistency, and quality control [109]. Therefore, future studies should evaluate antiviral efficacy together with pharmacokinetic, toxicological, formulation, manufacturing, and overall developability profiles before the clinical potential of marine-derived antiviral candidates can be adequately assessed. Nonclinical safety evaluation is also an essential component of progression toward clinical development.

### 8.2. Emerging Technologies for Marine-Derived Antiviral Discovery

Building on LC–MS/MS-based dereplication and molecular networking approaches, metabolomics-based strategies can facilitate the detection, comparison, and prioritization of chemically distinct metabolites from marine natural-product extracts for subsequent isolation and biological evaluation [100,101,103,110]. Artificial intelligence (AI) and machine-learning approaches may further complement these workflows by assisting in the prioritization and prediction of potentially bioactive marine natural products [111]. However, experimental validation remains essential.

Glycomics and complementary structural glycan analyses may further improve the characterization of sulfated glycans and their structure–activity relationships [112,113]. In particular, detailed characterization of sulfation patterns may help clarify how structural features influence interactions between sulfated glycans and viral proteins [113].

In addition, cryo-electron microscopy and AlphaFold-based structural prediction can provide complementary structural information on viral proteins and putative biomolecular interactions, thereby supporting mechanism-guided hypothesis generation, target characterization, and subsequent experimental validation [114,115,116]. Integration of these emerging technologies with conventional natural-product isolation, biological evaluation, and biochemical validation may therefore accelerate the discovery and mechanistic characterization of marine-derived antiviral candidates.

## 9. Conclusions

Evidence regarding marine-derived anti-flaviviral agents remains concentrated on DENV and ZIKV and spans two broad chemical domains. (1) sulfated algal macromolecules with reproducible early-stage activity and (2) chemically defined small molecules from algae and marine microorganisms with post-entry or replication-associated effects. The proposed dual-axis framework separates the implicated life cycle stage from the strength of mechanistic evidence, thereby avoiding the common conflation of time-of-addition data with direct target validation.

In the case of DENV, the inhibition of adsorption and internalization by sulfated polysaccharides represents the most consistent stage-resolved evidence, supporting the continued assessment of marine-derived polyanionic macromolecules as entry-directed antiviral probes. In the case of ZIKV, a more diverse range of diterpenes, alkaloids, cyclodepsipeptides, phlorotannins, and fungal metabolites have been reported, including several candidates with low-micromolar potency and favorable selectivity. However, most of these metabolites are supported primarily by cellular, virological, or stage-associated assays rather than by definitive molecular target evidence. Therefore, computationally proposed binders should remain clearly separated from experimentally validated antiviral agents.

These findings suggest that the limited number of studies on marine-derived anti-flaviviral agents should not necessarily be interpreted as evidence of weak antiviral potential. Rather, the current literature is characterized by limited mechanistic development and uneven experimental validation. Subsequent research should move beyond descriptive extract screening and focus on purified compound confirmation; standardized EC_50_, CC_50_, and SI testing; stage-resolved virological assays; and direct validation of conserved flaviviral targets, such as NS2B–NS3 protease, NS3 helicase, and NS5 RNA-dependent RNA polymerase.

JEV-specific evidence remains insufficient to define established marine-derived leads. However, the literature on DENV and ZIKV provides a practical basis for JEV-oriented natural product discovery. Integrating infectious virus screening, replicon- and target-based assays, LC-MS/MS dereplication, molecular networking, and genome-informed prioritization may accelerate the transition from preliminary extract-level activity to chemically defined and mechanistically interpretable antiviral candidates.

## Figures and Tables

**Table 1:** Stage-of-action classification of marine-derived anti-flaviviral agents.

Stage of Action	Evidence	Representative Agents	Virus	Ref.
Attachment	Adsorption inhibition	dl-galactan hybrids; carrageenans; *D. menstrualis* F-6 fraction	DENV and ZIKV	[20,28,46]
Internalization/uncoating	Internalization inhibition	λ-/ι-carrageenans	DENV	[28]
Virucidal	Virion infectivity loss	(4*R*,9*S*,14*S*)-4α-acetoxy-9β,14α-dihydroxydolast-1(15),7-diene; *D. menstrualis* FAc-2 fraction	ZIKV	[30,46]
Post-entry/replication	Post-entry activity; reduced viral markers	Marine-derived bacterial metabolites; *Fusarium* indole alkaloids; *Beauveria* cyclodepsipeptides; Dieckol	DENV and ZIKV	[31,32,48,49]
Post-entry/ productive infection, unresolved	Stage unresolved	unresolved	DENV	[44]

**Table 2:** Evidence levels for marine-derived anti-flaviviral agents.

Evidence Tier	Evidence Type	Interpretation	Representative Agents	Ref.
E1	Endpoint antiviral activity without adequate selectivity	Preliminary cellular or extract-level signal	Seaweed/microalgal extracts	[50]
E2	Dose–response activity with cytotoxicity and/or selectivity assessment	Validated cellular activity, stage unresolved	Fusarium indole alkaloids; scequinadoline A microbial butyrolactones/DKPs	[31,44,48]
E3	Adsorption, internalization, virucidal, or time-of-addition assays	Probable stage-of-action evidence	Carrageenans; dl-galactans; (4*R*,9*S*,14*S*)-4α-acetoxy-9β,14α-dihydroxydolast-1(15),7-diene; *D. menstrualis* fractions; *B. triquetrum* extract	[20,28,30,46,47]
E4	Concordant orthogonal viral RNA, viral protein, viral titer, or infectious-yield readouts	Orthogonal virological evidence	*Beauveria* cyclodepsipeptides; *E. cava*/dieckol	[32,49]
E5	Direct biochemical, biophysical, or genetic target validation	Defined target engagement	Harzianopyridone–RdRp	[33]
C	Computational prediction only; no experimental validation	Computational evidence only	Marine brown-algae-derived JEV RdRp candidates	[51]

**Table 3:** Representative marine-derived anti-flaviviral agents classified by antiviral activity, assay evidence, stage of action, and evidence strength.

Material	Virus	Activity	Evidence	Stage	Key Assay	Mechanism Type	Main Limitation	Ref.
dl-galactan	DENV	0.19–1.7 µg/mL ^a^;	E3	Attachment	Plaque + adsorption	Virus-directed	No target validation	[20]
λ-/ι-carrageenans	DENV	0.14–4.1 µg/mL ^b^; >1000 µg/mL ^c^; 244–6666 ^d^	E3	Entry	Adsorption/ internalization; RNA transfection	Virus-directed	Serotype/cell-dependent	[28]
*D. menstrualis* F-6	ZIKV	2.80 µg/mL ^b^; 592 µg/mL ^c^; ~211 ^d^	E3	Adsorption	Infection + adsorption	Virus-directed	Active constituent unknown	[46]
*D. menstrualis* FAc-2	ZIKV	0.81 µg/mL ^b^; 482 µg/mL ^c^; 595.06 ^d^	E3	Virucidal	Infection + virucidal	Virus-directed	Fraction-level evidence	[46]
(4*R*,9*S*,14*S*)-4α-acetoxy-9β,14α-dihydroxydolast-1(15),7-diene	ZIKV	0.75–0.95 µM ^b^; 935 µM ^c^; ~1246 ^d^	E3	Virucidal/ post-entry	Replication + virucidal + time-of-addition	Virus-directed	Target unknown	[30]
*B. triquetrum* extract	ZIKV	1.38 µg/mL ^b^; 400 µg/mL ^c^; 289.85 ^d^	E3	Post-entry	Plaque + virucidal + time-of-addition	Unresolved	Multiple actives possible	[47]
Marine-derived bacterial metabolites	DENV	11.2–21.2 µM ^b^	E2	Replication	Cell-based replication model	Unresolved	Target not validated	[48]
*Fusarium* indole alkaloids	ZIKV	4.2–7.5 µM ^b^	E2	Unresolved	Plaque reduction + cytotoxicity	Unresolved	Entry/target assays unavailable	[31]
*Beauveria* cyclodepsipeptides	ZIKV	10 µM ^e^; >100 µM ^c^	E4	RNA/ protein-associated	ZIKV RNA + NS5 protein	Unresolved	Compound-specific potency incomplete	[32]
Dieckol	ZIKV	4.88 µM ^a^; >100 µM ^c^	E4	Replication/hostmodulatory	Titer + mRNA + inflammatory markers	Potentially host-modulatory	Direct viral target not established	[49]
*E. cava* extract	ZIKV	NA	E4	Replication/hostmodulatory	Titer + mRNA + inflammatory markers	Potentially host-modulatory	Active constituent and direct viral target not established	[49]
*Tetraselmis* sp. chlorophyll extracts	ZIKV	NA	E1	Productive infection	Cell-based infection model	Unresolved	Active constituent/stage unresolved	[50]
Harzianopyridone	ZIKV	0.46–2.63 µM ^b^; >45 µM ^c^	E5	RdRp-associated	Cell-based inhibition + RdRp assays	Virus-directed (RdRp)	Further resistance/mutational validation desirable	[33]
Marine brown algae-derived JEV RdRp candidates	JEV	Not tested	C	RdRp hypothesized	Docking + molecular dynamics	Computationally proposed virus-directed	Computational only; not experimentally validated	[51]

Notes: ^a^ IC_50_, half-maximal inhibitory concentration; ^b^ EC_50_, half-maximal effective concentration; ^c^ CC_50_, half-maximal cytotoxic concentration; ^d^ SI, selectivity index, ^e^ tested concentration, with no EC_50_ or IC_50_ value reported.

**Table 4:** Contextual examples of marine-derived anti-flaviviral agents (not a rank-order potency comparison).

Virus	Origin	Representative Agents	Evidence Level	Source	Reported Activity	Mechanistic Note	Ref.
ZIKV	Marine	(4*R*,9*S*,14*S*)-4α-acetoxy-9β,14α-dihydroxydolast-1(15),7-diene	E3	Brown alga derived diterpene	0.75–0.95 µM ^b^; 935 µM ^c^; ~1246 ^d^	Antiviral activity Mechanism unclear	[30]
ZIKV	Marine	Harzianopyridone	E5	Marine-derived fungal Natural product	0.46–2.63 µM ^b^; >45 µM ^c^	RdRp-associated inhibition	[33]
ZIKV	Marine	Dieckol	E4	Brown alga- derived phlorotannin	4.88 µM ^a^; >100 µM ^c^	ZIKV inhibition; mechanism unclear	[49]
DENV	Marine	Scequinadoline A	E2	Marine Fungus	50 µM ^e^	DENV inhibition; mechanism unclear	[44]
ZIKV	Marine	JBIR-03	E2	Marine Fungus	4.2 µM ^b^	Not Validated	[31]
ZIKV	Marine	Destruxin A	E4	Marine fungus	10 µM ^e^; >100 µM ^c^	Reduced ZIKV RNA and NS5 protein levels	[32]
ZIKV	Marine	Fucoxanthin	E4	Brown alga derived carotenoid	12.5, 25, 50 µM ^g^	Docking-supported; target validation needed	[45]
JEV	Marine	Carrageenan	E3	Red seaweed-derived sulfated polysaccharide	15 µg/mL ^b^; >200 µg/mL ^c^; >13.3 ^d^	Inhibition of viral attachment and cellular entry	[72]
JEV	Marine	*Ulva lactuca* sulfated polysaccharide extract	E3	Green alga	NA	Inhibition of viral adsorption/entry	[71]
JEV	Marine	Sargothunbergol A	C	*Sargassum thunbergii*	No experimental; computational only	JEV NS5 RdRp binding proposed	[51]
JEV	Marine	Amentadione	C	*Carpodesmia amentacea*	No experimental; computational only	JEV NS5 RdRp binding proposed	[51]
JEV	Marine	Cystone A	C	*Cystoseira usneoides*	No experimental; computational only	JEV NS5 RdRp binding proposed	[51]
JEV	Marine	Cystodione C	C	*Cystoseira usneoides*	No experimental; computational only	JEV NS5 RdRp binding proposed	[51]

Notes: ^a^ IC_50_, half-maximal inhibitory concentration; ^b^ EC_50_, half-maximal effective concentration; ^c^ CC_50_, half-maximal cytotoxic concentration; ^d^ SI, selectivity index; ^e^ tested concentration, with no EC_50_ or IC_50_ value reported; ^g^ concentrations tested in the antiviral assay, with no fitted EC_50_ or IC_50_ value reported.

**Table 5:** Proposed assay-driven workflow for JEV-oriented marine-derived natural product discovery.

Step	Assay or Workflow Component	Purpose
1	Infectious JEV cell-based primary screen	Identify extracts/fractions/compounds that suppress infection
2	Cytotoxicity counter-screen	Remove nonspecific toxic or cytostatic samples
3	EC_50_, CC_50_, and SI Determination	Prioritize potent and selective candidates
4	Plaque/focus-forming assay, RT-qPCR, and viral protein detection	Confirm suppression of productive infection
5	Time-of-addition, attachment, entry, and virucidal assays	Classify stage of action
6	Replicon/ subgenomic RNA assay	Separate replication effects from entry and release
7	NS2B-NS3, NS3 helicase, and NS5 RdRp assays	Validate viral target engagement
8	LC–MS/MS dereplication and molecular networking	Avoid known/nonspecific metabolites and prioritize novelty
9	Isolation, structural elucidation and retesting	Confirm true active principle
10	PK, CNS exposure, in vivo efficacy, and toxicity	Assess translational feasibility for neuroinvasive infection

## Data Availability

No new data were created or analyzed in this study. Data sharing is not applicable to this article.

## Abbreviations

The following abbreviations are used in this manuscript:

DENV	dengue virus
ZIKV	Zika virus
JEV	Japanese encephalitis virus
RdRp	RNA-dependent RNA polymerase
NS5	nonstructural protein 5
CPE	cytopathic effect
SAR	structure–activity relationship
NS2B-NS3	nonstructural protein 2B–nonstructural protein 3
E1	Endpoint antiviral activity without compound-level or stage-resolved support
E2	reproducible dose–response activity with cytotoxicity or selectivity information
E3	stage-specific experimental evidence
E4	orthogonal virological evidence
E5	direct biochemical, biophysical, or genetic target validation
C	computational prediction only
ELISA	enzyme-linked immunosorbent assay
SI	selectivity index, CC_50_/EC_50_
PFU	plaque-forming unit
TNF-α	tumor necrosis factor-alpha
IFIT	interferon-induced protein with tetratricopeptide repeats
NS3	nonstructural protein 3
CNS	central nervous system
RT-qPCR	reverse transcription quantitative polymerase chain reaction
FAc	ethyl acetate fraction
EC_50_	Half maximal effective concentration
IC_50_	Half maximal inhibitory concentration
CC_50_	Half maximal cytotoxic concentration
